# Exclusive Breastfeeding and Growth Trajectories Until 5 Years of Age Among Children Monitored in Primary Health Care in Brazil

**DOI:** 10.1002/ajhb.70315

**Published:** 2026-07-20

**Authors:** Andressa Freire Salviano, Bianca de Melo Guedes, Luana Monteiro Barros, Nathália Teixeira de Oliveira, Dayana Rodrigues Farias, Bárbara Hatzlhoffer Lourenço

**Affiliations:** ^1^ Public Health Nutrition Program, School of Public Health, University of São Paulo, São Paulo São Paulo Brazil; ^2^ Department of Social and Applied Nutrition, Nutritional Epidemiology Observatory, Institute of Nutrition Josue de Castro Federal University of Rio de Janeiro, Rio de Janeiro Rio de Janeiro Brazil; ^3^ Department of Nutrition, School of Public Health University of São Paulo, São Paulo São Paulo Brazil

**Keywords:** child growth, exclusive breastfeeding, longitudinal studies, primary health care

## Abstract

**Objectives:**

Exclusive breastfeeding (EBF) is relevant for child growth, especially in contexts of socioeconomic vulnerability. This study estimates growth trajectories until 5 years of age according to EBF among children monitored in primary health care in Brazil.

**Methods:**

Longitudinal analysis using data from the Food and Nutrition Surveillance System (2015–2019). EBF was characterized according to food intake markers in two age groups (0 to < 3 and 3 to < 6 months). Mixed linear models with restricted cubic splines estimated trajectories of BMI‐for‐age (BAZ; *n* = 148 705 at 0 to < 3 months, *n* = 135 662 at 3 to < 6 months) and height‐for‐age (HAZ; *n* = 127 812 at 0 to < 3 months, *n* = 108 846 at 3 to < 6 months) z‐scores using repeated anthropometric measurements collected after the exposure assessment.

**Results:**

BAZ and HAZ were significantly higher until 6 months among exclusively breastfed children. Between ages 9–36 months, EBF interruption at 0 to < 3 months was associated with higher BAZ (reaching 0.70 z, 95% CI 0.69; 0.72 at age 18 months, vs. EBF: 0.59 z, 95% CI 0.58; 0.60). Higher HAZ was observed after age 9 months among children who were not exclusively breastfed. EBF interruption at 0 to < 3 months was related to higher BAZ at age 60 months (0.62 z, 95% CI 0.49; 0.74) compared to interruption at 3 to < 6 months (0.36 z, 95% CI 0.29; 0.44).

**Conclusions:**

EBF was associated with higher BAZ and HAZ until age 6 months. Early EBF interruption led particularly to higher BAZ trajectories until age 5 years. Promoting adequate infant feeding practices may improve child growth.

## 1 Introduction

Breastfeeding (BF) is one of the most effective interventions for child health promotion in early years. When exclusive in the first 6 months, it is associated with reduction in infant mortality and the risk of infectious and respiratory diseases, in addition to improving motor and cognitive skills (Dib et al. 2024; Horta et al. 2023; Victora et al. 2016). However, recent estimates showed that only 48.6% of children under 6 months of age in 86 low‐ and middle‐income countries (LMIC) were on exclusive BF (EBF) (Neves et al. 2021), which is off‐track of the Sustainable Development Goals (SDG) of 70% of EBF by 2030 (United Nations [UN] 2020).

Considering the complex dynamics of malnutrition, inadequate BF practices are concerning, especially in contexts of greater socioeconomic vulnerability. Between 2000 and 2024, the average prevalence of overweight increased among children below 5 years, particularly in upper‐middle‐income countries (6.7%–8.6%). Despite reductions in stunting, substantial occurrence was observed in low‐ (35.8%) and lower‐middle‐income countries (28.9%) in 2024 (United Nations Children's Fund [UNICEF], World Health Organization [WHO], World Bank Group [WBG] 2025).

Previous studies have indicated that BF practices are associated with growth trajectories during infancy. Among 595 children in the United States, those who were breastfed for less than 2 months were more likely to follow an increasing weight gain trajectory than their counterparts (OR: 2.55, 95% CI 1.14; 5.72) (Carling et al. 2015). Formula‐fed infants had higher BMI‐for‐age z‐scores (BAZ) at age 7 months (difference: 0.35, 95% CI 0.00; 0.69) than predominantly breastfed infants, whereas length‐for‐age z‐scores were similar between groups (Bell et al. 2017). These findings are consistent with a meta‐analysis of 159 observational studies and clinical trials showing that EBF protected against overweight and obesity during childhood and adolescence (pooled OR: 0.68, 95% CI 0.62; 0.74) (Horta et al. 2023).

An analysis based on 33 cohorts conducted in LMIC estimated that exclusive or predominant BF would increase length‐for‐age z‐scores by 0.03 at 6 months (95% CI 0.01; 0.04), although this effect was not sustained at 24 months of age (0.01 z, 95% CI −0.00; 0.02) (Mertens et al. 2023). Considering that the highest incidence of stunting occurs between 3 and 6 months of age (Benjamin‐Chung et al. 2023), understanding the relationship between EBF and growth trajectories during this critical period remains particularly relevant.

Investing in optimal child growth through the promotion of effective EBF offers economic, environmental, and health benefits, and is especially advantageous in vulnerable contexts (Rollins et al. 2016). In this sense, robust nutritional data and well‐structured information systems are essential to support ongoing policies and research (Heidkamp et al. 2021). In Brazil, food and nutrition surveillance (FNS) in primary health care (PHC) includes food intake markers and serial anthropometric data, covering the most socioeconomically vulnerable children (Campos and Fonseca 2021). These routinely collected data provide an opportunity to evaluate whether EBF indicators as assessed through FNS are associated with subsequent child growth, supporting population‐level monitoring and public health decision‐making.

This study aimed to analyze growth trajectories up to 5 years of age according to EBF in the first 6 months among children monitored in PHC countrywide. We additionally sought to understand whether different timing of exposure to EBF interruption was associated with variations in child growth trajectories.

## 2 Materials and Methods

### 2.1 Study Design and Data Source

This longitudinal study used data from children under 5 years of age available in the Brazilian Food and Nutrition Surveillance System (SISVAN) from 2015 to 2019. The study was approved by the Research Ethics Committee of the School of Public Health of the University of São Paulo (number 4.172.787/2020) and conducted according to the Declaration of Helsinki guidelines. All secondary data were anonymous and informed consent from subjects was not necessary.

SISVAN consolidates information on food intake markers and nutritional status of the population monitored in PHC in Brazil, mainly covering socioeconomically vulnerable children (Campos and Fonseca 2021). The data are routinely collected by health professionals using standardized protocols (Brazil 2023). Recent findings indicate stable EBF rates among children monitored in PHC, with a prevalence of 54.5% in 2019, still below the SDG target of 70% by 2030 (Salviano et al. 2024).

**FIGURE 1:**
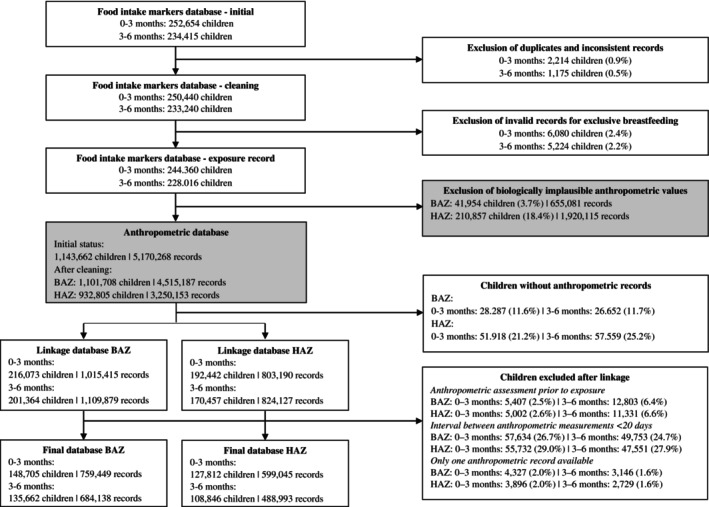
Selection of children monitored in Primary Health Care with information on exclusive breastfeeding and nutritional status, Brazil 2015–2019.

Food intake markers in children under 6 months and anthropometric measurements up to 5 years were analyzed from 2015 to 2019. Datasets were cleaned to exclude duplicates, inconsistent ages, and children recorded in different municipalities. Food intake and anthropometric information were linked using a unique identification number provided by the Brazilian Ministry of Health (Figure 1).

### 2.2 Measurements

#### 2.2.1 Assessment of Exclusive Breastfeeding

Food intake markers for children under 6 months of age included nine items with answers regarding the previous day (no/yes), for the screening of infant feeding practices (i.e., intake of water, tea, infant formula and other milk, and consumption of solid foods) (Brazil 2023; Salviano et al. 2024). EBF was classified as “yes” (positive answer to breast milk only, without other liquids or solids) or “no” (positive responses to water/tea or infant formula or other milks or any complementary solid food, whether or not associated with breast milk).

We considered two age groups to explore exposure to EBF in critical periods of child feeding patterns and development (Benjamin‐Chung et al. 2023; Mertens et al. 2023; Victora et al. 2016): 0 to < 3 months and 3 to < 6 months. For the assessment in each of these age groups, the exposure to EBF was defined according to the first valid record for each child, i.e., a complete set of answers to food intake markers to characterize EBF.

#### 2.2.2 Anthropometric Data

Information on child's weight and length/height was used to calculate sex‐specific BAZ and length/height‐for‐age z‐scores (HAZ) according to the WHO child growth standards (WHO 2006), with the aid of the zanthro package in Stata version 17 (2023).

Biologically implausible values for nutritional status, as defined by WHO for BAZ (< −6 and > 5 z‐scores) and HAZ (< −6 or > 6 z‐scores) (WHO and UNICEF 2019), were excluded. For HAZ, we also removed children showing a reduction in length/height greater than −2 cm between two assessments. This step was necessary as data were produced in different PHC services. Outliers in growth trajectories were identified through studentized residuals estimated from linear mixed models with restricted cubic splines for age, as previously described (Boone‐Heinonen et al. 2019). Records with residuals outside ±2 standard deviations were excluded. For BAZ, implausible weight and length/height values were removed before data cleaning.

Plausible repeated BAZ and HAZ measurements up to 5 years of age following exposure assessment and at a minimum interval of 20 days were considered, in order to reduce potential measurement error (Wright et al. 2022). Children assessed only once for the outcomes were not included in the analysis.

#### 2.2.3 Covariates

Child's sex (female or male) and municipal‐level information on human development index (HDI), and the Brazilian Early Childhood Friendly Municipal Index (IMAPI) were considered as covariates. The HDI varies from 0 to 1 according to the dimensions of longevity, education and income, and was categorized as low (up to 0.550), medium (between 0.551 and 0.699), high (between 0.700 and 0.799), or very high (above 0.800) (Instituto Brasileiro de Geografia e Estatística [IBGE] 2012). Based on the Nurturing Care (NC) framework, IMAPI assessed the provision of a timely environment for early childhood development at a local level in Brazil according to 31 indicators. Categorized into quintiles, the highest and lowest fifths of IMAPI correspond to the best and worst conditions of NC, respectively (Buccini et al. 2022).

### 2.3 Data Analysis

Normality of BAZ and HAZ distributions was confirmed using the Kolmogorov–Smirnov test and graphical inspection. For each analytical sample (BAZ and HAZ), baseline sociodemographic and contextual characteristics were compared according to exposure to EBF at 0 to < 3 and 3 to < 6 months, using the *t*‐test or chi‐square test for continuous or categorical variables, respectively.

Growth trajectories according to EBF were estimated using mixed linear models with restricted cubic splines. Cubic splines are non‐linear terms that allow smoothing the relationship between anthropometric indices and age (Durrleman and Simon 1989; Lourenço et al. 2015). Piecewise cubic polynomials were joined at knots at ages 2, 6, 12, 24, and 48 months, important reference points in WHO growth curves (WHO 2006) and early childhood growth faltering (Benjamin‐Chung et al. 2023; Mertens et al. 2023).

Initially, models included the outcome (BAZ or HAZ), EBF exposure (assessed at 0 to < 3 months or 3 to < 6 months), child's sex, linear and spline terms for age in months, and interaction terms between EBF and age. Random effects for the intercept and linear term for age (slope) were included to account for within‐person correlation in variance estimation (Diggle et al. 2002), with the specification of an unstructured covariance matrix. The number and timing of outcome measurements were allowed to vary across participants, and all available measurements were included (Diggle et al. 2002). For sensitivity analysis, additional models included a municipality‐level random intercept (IBGE municipality code) to account for potential clustering at this level.

Final models were additionally adjusted for municipal HDI and IMAPI. Potential cohort effects were tested by including year of birth, but this variable was excluded as the results remained unchanged. The lowest Akaike Information Criterion (AIC) and Bayesian Information Criterion (BIC) values were also used to select the best‐fitting models. Predicted BAZ and HAZ values and 95% confidence intervals (95% CI) were estimated every 3 months in the first year and at 6‐month intervals up to 5 years of age according to EBF. Analyses were performed in Stata version 17 (StataCorp, College Station, TX, USA).

## 3 Results

At baseline, considering EBF assessment at 0 to < 3 months of age (49.2% girls; mean [SD] age: 1.3 [0.8] months), 148 705 children had information for BAZ (mean: 0.29 [1.21] z; 7.4% overweight) and 127 812 children for HAZ (mean: 0.12 [1.51] z; 7.5% stunted). Regarding EBF assessment at 3 to < 6 months (49.2% girls; mean age: 4.2 [0.8] months), data on BAZ were available for 135 662 children (mean: 0.20 [1.20] z; 6.7% overweight), while 108 846 children had HAZ measurements (mean: 0.01 [1.36] z; 6.6% stunted).

Regarding baseline characteristics, children exposed to EBF were slightly younger and had higher baseline mean BAZ and HAZ values. Small differences in sex distribution were observed. Exclusively breastfed children were more likely to reside in municipalities with higher HDI and IMAPI levels (Table 1).

**TABLE 1:** Baseline characteristics of Brazilian children monitored in Primary Health Care according to exposure to exclusive breastfeeding at 0 to < 3 or 3 to < 6 months of age, considering analyses for body mass index‐for‐age z‐score (BAZ) and height‐for‐age z‐score (HAZ).

	Children included in the BAZ analysis						Children included in the HAZ analysis					
	EBF^b^ at 0 to < 3 months *n* = 148 705			EBF^b^ at 3 to < 6 months *n* = 135 662			EBF^b^ at 0 to < 3 months *n* = 127 812			EBF^b^ at 3 to < 6 months *n* = 108 846		
	Yes	No	*p* ^a^	Yes	No	*p* ^a^	Yes	No	*p* ^a^	Yes	No	*p* ^a^
BAZ^c^	0.33 (1.18)	0.20 (1.25)	< 0.001	0.22 (1.18)	0.20 (1.21)	0.005						
HAZ^c^							0.18 (1.46)	−0.02 (1.61)	< 0.001	0.04 (1.31)	−0.01 (1.40)	< 0.001
Age^d^	1.21 (0.82)	1.60 (0.80)	< 0.001	3.97 (0.75)	4.35 (0.85)	< 0.001	1.19 (0.82)	1.59 (0.80)	< 0.001	3.97 (0.75)	4.35 (0.85)	< 0.001
Sex			< 0.001			< 0.001			< 0.001			< 0.001
Female	49.9	47.9		50.2	48.6		49.8	47.7		50.1	48.6	
Male	50.1	52.1		49.8	51.4		50.2	52.3		49.9	51.4	
HDI^e^			< 0.001			< 0.001			< 0.001			< 0.001
Low	2.3	3.4		3.2	3.9		1.8	2.7		2.5	3.1	
Medium	36.3	42.2		42.7	46.5		33.4	38.6		39.0	42.6	
High	54.7	49.2		47.5	44.3		57.3	52.7		50.7	47.9	
Very high	6.7	5.2		6.6	5.3		7.5	6.0		7.8	6.4	
IMAPI^f^			< 0.001			< 0.001			< 0.001			< 0.001
Q1	21.5	26.2		23.0	28.1		21.2	25.6		22.1	27.5	
Q2	21.7	20.3		20.2	18.9		21.8	20.6		20.1	18.9	
Q3	21.6	21.7		21.2	21.3		21.6	21.6		21.5	21.2	
Q4	18.3	16.6		17.6	15.9		18.4	17.0		17.7	16.4	
Q5	16.9	15.2		18.0	15.8		17.0	15.2		18.6	16.0	

Abbreviations: BAZ: BMI‐for‐age z‐score; EBF: Exclusive breastfeeding; HAZ: height‐for‐age z‐score; HDI: Human Development Index; IMAPI: Brazilian Early Childhood Friendly Municipal Index; Q1: lowest quintile; Q2: second quintile; Q3: third quintile; Q4: fourth quintile; Q5: highest quintile.

^a^ Comparisons between exclusive breastfeeding groups for BAZ, HAZ and age: *t*‐test; for sex, HDI and IMAPI: *χ* ^2^ test.

^b^ Exclusive breastfeeding (EBF): yes (infants 0 to < 6 months of age with positive answer to breast milk only, without other liquids or solids); no (infants 0 to < 6 months of age with positive responses to water/tea or infant formula or other milks or any complementary solid food, whether or not associated with breast milk).

^c^ Mean (SD) of BAZ and HAZ in z‐scores.

^d^ Mean (SD) age in months.

^e^ HDI: Municipal‐level information on human development index. Categories: low (≥ 0.550), medium (between 0.551–0.699), high (between 0.700–0.799), and very high (≥ 0.800).

^f^ IMAPI: overall score of the Brazilian Early Childhood Friendly Municipal Index, classified in quintiles. The index is composed of the Nurturing Care domains of adequate nutrition, good health, opportunities for early learning, and security and safety.

Considering EBF assessment at 0 to < 3 months of age, the mean follow‐up period was 33.9 (25.2) months for BAZ (759 449 repeated measurements; median [IQR]: 4 [3–6] records per child; range: 2–44) and 29.5 (22.5) months for HAZ (599 045 repeated measurements; median [IQR]: 4 [3–6] records per child; range: 2–46). The median interval between consecutive measurements was 42 (30–73) days for both outcomes. For EBF assessment at 3 to < 6 months of age, the mean follow‐up period was 29.5 (22.5) months for BAZ (684 138 repeated measurements; median [IQR]: 4 [2–6] records per child; range: 2–43) and 29.5 (22.8) months for HAZ (488 993 repeated measurements; median [IQR]: 4 [2–6] records per child; range: 2–46). The median interval between consecutive measurements was 42 (30–77) days for BAZ and 43 (31–81) days for HAZ (Figure 1).

**FIGURE 2:**
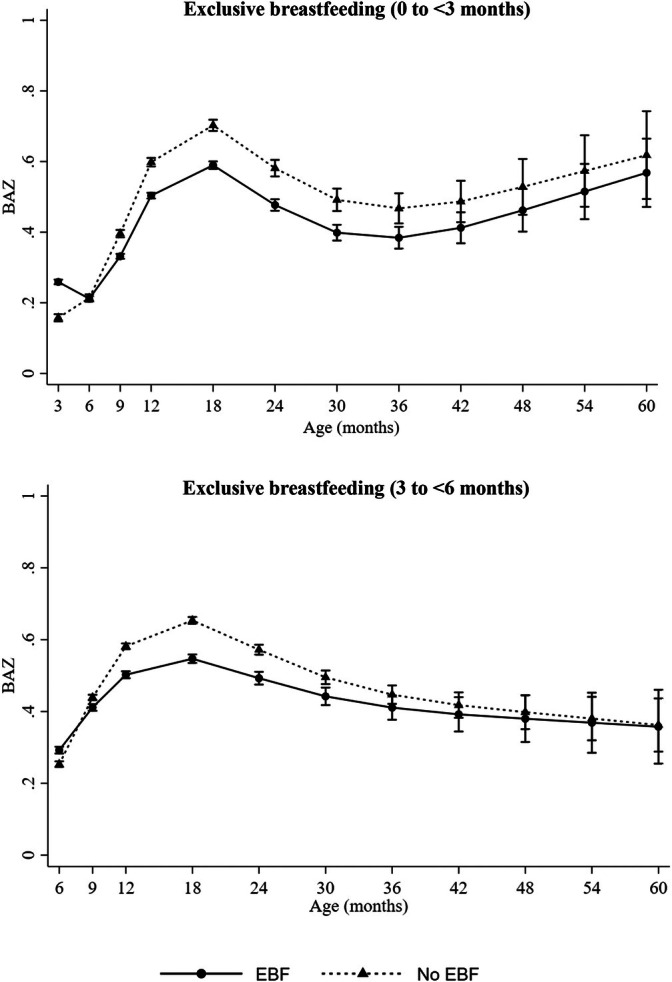
Body mass index‐for‐age z‐scores (BAZ) trajectories up to age 5 years according to exposure to exclusive breastfeeding in Brazilian children monitored in Primary Health Care, 2015–2019.

Tables 2 and 3 show unadjusted and adjusted analyses for BAZ and HAZ trajectories, respectively. Considering adjustment for municipal HDI and IMAPI, BAZ and HAZ trajectories between ages 3 and 6 months of age were higher for exclusively breastfed children compared to their counterparts, regardless of the age group for EBF assessment. Among children who interrupted EBF at 0 to < 3 months, higher mean BAZ was observed from ages 9 to 36 months, reaching 0.70 z (95% CI 0.69; 0.72) at age 18 months (in contrast to EBF: 0.59 z, 95% CI 0.58; 0.60). A similar pattern was observed among children who were assessed at 3 to < 6 months, but for a shorter period (ages 9 to 30 months) and with smaller differences in mean BAZ between EBF categories. At 5 years of age, BAZ trajectories did not differ according to EBF categories, but higher mean BAZ was associated with earlier EBF interruption (0 to < 3 months: 0.62 z, 95% CI 0.49; 0.74, and 3 to < 6 months: 0.36 z, 95% CI 0.29; 0.44) (Table 2, Figure 2).

**TABLE 2:** Body mass index‐for‐age z‐scores (BAZ) up to age 5 years according to exposure to exclusive breastfeeding in Brazilian children monitored in Primary Health Care, 2015–2019.

	Mean BAZ (95% CI) according to exclusive breastfeeding			
	Unadjusted model^b^		Adjusted model^c^	
	Exclusive breastfeeding^a^		Exclusive breastfeeding^a^	
Age in months	Yes	No	Yes	No
Exposure 0 to < 3 months (*n* = 148 705)				
3	0.25 (0.24; 0.26)	0.16 (0.15; 0.17)	0.26 (0.25; 0.27)	0.16 (0.15; 0.17)
6	0.20 (0.19; 0.21)	0.22 (0.21; 0.23)	0.21 (0.20; 0.22)	0.21 (0.20; 0.22)
9	0.32 (0.31; 0.33)	0.41 (0.40; 0.42)	0.33 (0.32; 0.34)	0.40 (0.39; 0.41)
12	0.50 (0.49; 0.51)	0.61 (0.60; 0.62)	0.50 (0.49; 0.51)	0.60 (0.59; 0.61)
18	0.58 (0.57; 0.59)	0.71 (0.70; 0.73)	0.59 (0.58; 0.60)	0.70 (0.69; 0.72)
24	0.47 (0.46; 0.49)	0.59 (0.57; 0.62)	0.48 (0.46; 0.49)	0.58 (0.56; 0.60)
36	0.38 (0.35; 0.41)	0.48 (0.44; 0.52)	0.38 (0.35; 0.41)	0.47 (0.42; 0.51)
48	0.46 (0.40; 0.52)	0.54 (0.46; 0.62)	0.46 (0.40; 0.52)	0.53 (0.45; 0.61)
60	0.56 (0.47; 0.66)	0.63 (0.50; 0.75)	0.57 (0.47; 0.66)	0.62 (0.49; 0.74)
Exposure 3 to < 6 months (*n* = 135 662)				
6	0.28 (0.27; 0.29)	0.25 (0.24; 0.26)	0.29 (0.28; 0.30)	0.25 (0.24; 0.26)
9	0.40 (0.39; 0.41)	0.44 (0.43; 0.45)	0.41 (0.40; 0.42)	0.44 (0.43; 0.45)
12	0.49 (0.48; 0.50)	0.58 (0.57; 0.59)	0.50 (0.49; 0.51)	0.58 (0.57; 0.59)
18	0.53 (0.52; 0.55)	0.66 (0.65; 0.67)	0.55 (0.54; 0.56)	0.65 (0.64; 0.66)
24	0.48 (0.46; 0.50)	0.58 (0.56; 0.59)	0.49 (0.47; 0.51)	0.57 (0.56; 0.59)
36	0.40 (0.37; 0.43)	0.45 (0.42; 0.48)	0.41 (0.38; 0.44)	0.45 (0.42; 0.47)
48	0.37 (0.30; 0.43)	0.40 (0.35; 0.45)	0.38 (0.31; 0.44)	0.40 (0.35; 0.45)
60	0.35 (0.24; 0.45)	0.36 (0.29; 0.44)	0.36 (0.25; 0.46)	0.36 (0.29; 0.44)

^a^ Exclusive breastfeeding (EBF): yes (infants 0 to < 6 months of age with positive answer to breast milk only, without other liquids or solids); no (infants 0 to < 6 months of age with positive responses to water/tea or infant formula or other milks or any complementary solid food, whether or not associated with breast milk).

^b^ Unadjusted model: linear mixed model with restricted cubic splines for age, including BAZ as the outcome, EBF yes/no as the exposure, child's sex, linear and spline terms for age with knots at 2, 6, 12, 24, and 48 months, and interaction terms between EBF categories and age.

^c^ Adjusted model: adjusted for municipal‐level information on human development index (HDI) and the Brazilian Early Childhood Friendly Municipal Index (IMAPI).

**TABLE 3:** Height‐for‐age z‐scores (HAZ) up to age 5 years according to exposure to exclusive breastfeeding in Brazilian children monitored in Primary Health Care, 2015–2019.

	Mean HAZ (95% CI) according to exclusive breastfeeding			
	Unadjusted model^b^		Adjusted model^c^	
	Exclusive breastfeeding^a^		Exclusive breastfeeding^a^	
Age in months	Yes	No	Yes	No
Exposure 0 to < 3 months (*n* = 127 812)				
3	0.13 (0.12; 0.14)	−0.02 (−0.03; −0.01)	0.13 (0.12; 0.14)	−0.02 (−0.03; −0.01)
6	0.06 (0.05; 0.07)	0.05 (0.04; 0.06)	0.06 (0.05; 0.07)	0.05 (0.04; 0.07)
9	−0.03 (−0.04; −0.02)	0.03 (0.01; 0.04)	−0.03 (−0.04; −0.02)	0.03 (0.02; 0.04)
12	−0.12 (−0.13; −0.11)	−0.04 (−0.06; −0.03)	−0.12 (−0.13; −0.11)	−0.04 (−0.05; −0.02)
18	−0.20 (−0.21; −0.18)	−0.11 (−0.13; −0.09)	−0.20 (−0.21; −0.18)	−0.11 (−0.13; −0.09)
24	−0.18 (−0.20; −0.15)	−0.10 (−0.13; −0.06)	−0.18 (−0.20; −0.15)	−0.09 (−0.13; −0.06)
36	−0.06 (−0.11; −0.01)	0.08 (0.02; 0.15)	−0.06 (−0.11; −0.01)	0.09 (0.02; 0.15)
48	0.10 (−0.01; 0.20)	0.36 (0.23; 0.50)	0.09 (−0.01; 0.19)	0.37 (0.23; 0.50)
60	0.26 (0.09; 0.43)	0.66 (0.45; 0.88)	0.25 (0.09; 0.42)	0.66 (0.45; 0.88)
Exposure 3 to < 6 months (*n* = 108 846)				
6	0.03 (0.02; 0.04)	0.01 (0.00; 0.02)	0.03 (0.02; 0.04)	0.01 (0.00; 0.02)
9	−0.05 (−0.06; −0.04)	−0.02 (−0.03; −0.01)	−0.06 (−0.07; −0.05)	−0.01 (−0.02; 0.00)
12	−0.13 (−0.14; −0.11)	−0.05 (−0.06; −0.04)	−0.13 (−0.14; −0.12)	−0.04 (−0.05; −0.03)
18	−0.21 (−0.23; −0.20)	−0.11 (−0.13; −0.10)	−0.22 (−0.23; −0.20)	−0.11 (−0.12; −0.10)
24	−0.24 (−0.26; −0.21)	−0.15 (−0.17; −0.13)	−0.24 (−0.26; −0.21)	−0.15 (−0.17; −0.13)
36	−0.13 (−0.18; −0.08)	−0.03 (−0.07; 0.01)	−0.14 (−0.19; −0.09)	−0.03 (−0.07; 0.01)
48	0.07 (−0.03; 0.17)	0.25 (0.18; 0.33)	0.07 (−0.04; 0.17)	0.26 (0.18; 0.33)
60	0.29 (0.12; 0.45)	0.56 (0.44; 0.68)	0.29 (0.12; 0.45)	0.56 (0.44; 0.69)

^a^ Exclusive breastfeeding (EBF): yes (infants 0 to < 6 months of age with positive answer to breast milk only, without other liquids or solids); no (infants 0 to < 6 months of age with positive responses to water/tea or infant formula or other milks or any complementary solid food, whether or not associated with breast milk).

^b^ Unadjusted model: linear mixed model with restricted cubic splines for age, including HAZ as the outcome, EBF yes/no as the exposure, child's sex, linear and spline terms for age with knots at 2, 6, 12, 24 and 48 months, and interaction terms between EBF categories and age.

^c^ Adjusted model: adjusted for municipal‐level information on human development index (HDI) and the Brazilian Early Childhood Friendly Municipal Index (IMAPI).

**FIGURE 3:**
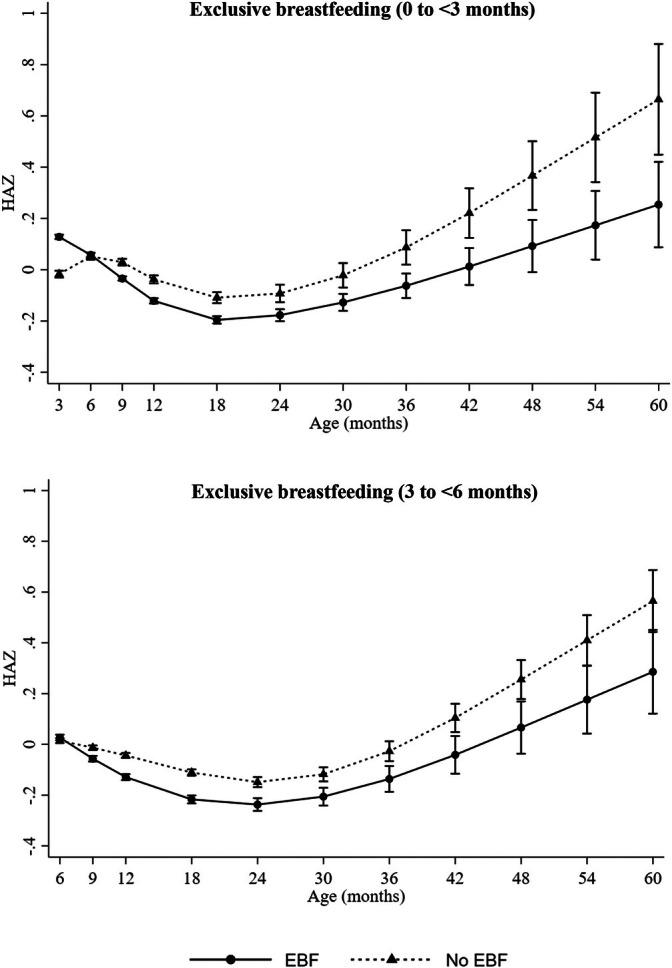
Height‐for‐age z scores (HAZ) trajectories up to age 5 years according to exposure to exclusive breastfeeding in Brazilian children monitored in Primary Health Care, 2015–2019.

For HAZ, a higher trajectory was observed between ages 9 and 60 months among children who interrupted EBF from 0 to < 3 months, reaching 0.66 z (95% CI 0.45; 0.88, compared to EBF: 0.25 z, 95% CI 0.09; 0.42). Considering EBF assessment at 3 to < 6 months, similar findings were observed from ages 9 to 54 months, with smaller differences in mean HAZ between EBF categories. Among children who were not exclusively breastfed, the highest mean HAZ was observed at age 54 months (0.41 z, 95% CI 0.31; 0.51, vs. EBF: 0.18 z, 95% CI 0.04; 0.30). At 5 years, HAZ trajectories did not differ by EBF categories nor considering the different timing in EBF assessment (Table 3, Figure 3).

In sensitivity analysis, municipality‐level intra‐class correlation coefficients ranged from 3.0% to 4.0%. Estimates for BAZ and HAZ according to EBF remained virtually unchanged with additional random effects at the municipality level, as presented in the Table S1.

## 4 Discussion

In this study, BAZ and HAZ trajectories were higher for exclusively breastfed children during the recommended period for this practice. From 9 months of age onwards, higher BAZ and HAZ trajectories were observed among children who interrupted EBF. Considering the timing of exposure (0 to < 3 months and 3 to < 6 months), our findings suggest that earlier EBF interruption may be concerning, as it was particularly associated with higher BAZ up to age 5 years.

The prevalence of EBF at 0 to < 3 months was 70.7% and declined to 40.8% at 3 to < 6 months among children monitored in PHC in Brazil. Estimates froms the Global Breastfeeding Scorecard indicate that EBF increased by 8 percentage points since 2020, reaching 47% in 2025 (WHO and UNICEF 2025), similar to estimates in LMIC (48.6% in 2019) (Neves et al. 2021). In Brazil, advances in EBF rates in children under 6 months were observed over time (26.9% in 1996, 39.0% in 2006, and 45.8% in 2019), but recent slowdowns in these trends had raised concerns (Boccolini et al. 2023). Strongly influenced by commercial determinants and sociocultural barriers, pre‐lacteal foods, such as water, teas, and formulas, often contribute to EBF interruption (Balogun et al. 2015). A meta‐analysis of 27 prospective studies associated the introduction of water‐ and milk‐based pre‐lacteal foods in the first weeks of life with an increased risk of EBF cessation in children under 6 months (RR 1.44, 95% CI 1.29; 1.60) (Pérez‐Escamilla et al. 2022).

In our study, EBF was associated with higher mean BAZ and HAZ during the first 6 months of life, after which non‐exclusively breastfed children showed higher growth trajectories. Because early EBF interruption encompasses a critical transition to complementary feeding with the potential to negatively influence child growth (Gingras et al. 2019; Gupta et al. 2025), some subsequent catch‐up in BAZ and HAZ could be expected. Nevertheless, evidence also suggests EBF plays a long‐term role in regulating weight gain and linear growth, which is consistent with the associations observed in our study population over the follow‐up period.

Regarding BAZ, a meta‐analysis of 10 population‐based birth cohorts showed that the introduction of complementary foods before 4 months, compared to 4–6 months, was associated with an increased risk of overweight (RR 1.18, 95% CI 1.06; 1.31) or obesity (RR 1.33, 95% CI 1.07; 1.64) in childhood (Wang et al. 2016). More recently, a systematic review of 9 cohorts from high‐income countries indicated that children who were exclusively or predominantly breastfed at 3–6 months had lower BMI or BAZ trajectories between 7 months and 8 years of age, compared to those who were fed infant formula or other food groups (Zheng et al. 2024). Our findings corroborate these results, given continuous BAZ consistently above the WHO standards, and suggest that the interruption of EBF at 0 to < 3 months may sustain higher trajectories for a longer period.

Several mechanisms elucidate the protective effects of EBF against higher BAZ. Compared to infant formula, breast milk contains lower protein levels and bioactive compounds that help prevent excessive weight gain (Pérez‐Escamilla et al. 2023). A systematic review of 10 European cohorts showed that excessive protein consumption in the first 2 years of life was associated with rapid weight gain, leading to higher mean BAZ and higher risk of childhood obesity (Stokes et al. 2021). BF promotes healthier intestinal microbiota, favoring the growth of beneficial bacteria such as Bifidobacterium and Lactobacillus. These stimulate the production of short‐chain fatty acids, responsible for inhibiting inflammatory cytokines and increasing the activity of regulatory immune cells (Petraroli et al. 2021). Bioactive substances in breast milk, such as leptin, insulin, glucagon‐like peptide‐1 and peptide YY, are associated with appetite regulation (Srivastava and Swati 2023). In a Brazilian birth cohort, a longer duration of BF (> 6 months) was shown to moderate the effect of FTO polymorphisms on adiposity at age 30 years (−0.23 cm in visceral fat thickness, 95% CI −0.41; −0.05) (Horta et al. 2018).

Concerning linear growth, EBF was associated with lower HAZ after age 9 months. Previous growth curves, which included mainly formula‐fed infants, showed a 6% difference in length between 1 and 4 months, compared to those in EBF (Dewey 1998; Nelson et al. 1989). Breast milk adjusts its protein composition to the child's growth needs, unlike formula (Verd et al. 2018). In a meta‐analysis of 19 randomised controlled trials, infants fed high‐protein formula (> 2.2 g/100 kcal) grew more than breastfed infants from 4 months onwards (0.06; 95% CI 0.04; 0.08) (Ren et al. 2022). Compared to breast milk, a high intake of dairy proteins may stimulate higher levels of insulin‐like growth factor 1, promoting faster growth (Putet et al. 2016).

In our study, mean HAZ values were below the median of the WHO growth standards during the first years of life, which is consistent with other studies in LMIC (Benjamin‐Chung et al. 2023; Roth et al. 2017; Stein et al. 2010). Our study population was composed of children monitored in PHC settings in Brazil, who are characterized by greater socioeconomic vulnerability (de Souza et al. 2023). In demographic and health surveys in LMIC between 1990 and 2017, milk consumption was associated with a 1.9% reduction (95% CI −0.02; −0.01) in stunting among children aged 6–59 months. However, this association may reflect better overall nutrition or higher socioeconomic status rather than indicating an advantage over EBF (Herber et al. 2020). Another study in 90 LMIC identified a positive correlation of per capita gross domestic product with infant formula consumption (*r* = 0.70, *p* < 0.0001) and a negative correlation with EBF (*r* = −0.37, *p* < 0.001) (Neves et al. 2020). Taken together, these results underline that structural, commercial, and sociocultural barriers to BF should be addressed through synergistic policy initiatives to promote, protect, and support BF (Baker et al. 2023) while preventing linear growth faltering.

Our study had some limitations. First, the use of secondary data may be subject to measurement bias, although there are rigorous protocols for FNS in PHC in Brazil. Second, the database had limited covariates on household socioeconomic characteristics and information on complementary feeding. Although the analyses were adjusted for municipal indicators to account for contextual differences, residual confounding due to unmeasured individual and family‐level factors cannot be ruled out. Additionally, race/skin color could not be adequately examined because of substantial missing data (21%), which is consistent with previous reports regarding Brazilian health information systems (Souza et al. 2024). Future studies should prioritize data collection and quality to properly investigate potential racial and ethnic disparities in child growth. However, the wide availability of EBF data allowed longitudinal analysis of growth trajectories with greater temporal coverage than previous studies in LMIC. Third, residual cohort effects may have resulted from EBF assessment in two age groups. Although groups differed in contextual variables at baseline, all children were followed between 2015 and 2019, and adjustment for year of birth did not change results. Analysis of different timing of BF exposure allowed a more detailed assessment of implications for child growth. As strengths, we highlight the robust use of FNS data in Brazil to longitudinally study BF practices in a socioeconomically vulnerable infant population.

In conclusion, exclusively breastfed children had higher BAZ and HAZ trajectories during the recommended EBF period. Early interruption of EBF was associated with higher BAZ and HAZ trajectories from 9 months onwards. Findings on HAZ trajectories should be interpreted with caution due to the association between early solid food introduction and weight gain, with higher BAZ by age 5 years, particularly in children not exclusively breastfed at 0 to < 3 months. Our results highlight the importance of promoting appropriate feeding practices from the first months of life to support optimal growth in vulnerable populations.

## Author Contributions

A.F.S. design, data analysis and interpretation, writing of the manuscript. B.M.G. and L.M.B. data management and interpretation. N.T.O. and D.R.F. data interpretation, critical review of the manuscript. B.H.L. conception and design, analysis supervision, data interpretation, writing and critical review of the manuscript. All authors approved the final version of the manuscript and are responsible for the submitted work.

## Funding

This work was supported by the Conselho Nacional de Desenvolvimento Científico e Tecnológico (Brazilian National Council for Scientific and Technological Development), grant number 442963/2019‐0.

## Ethics Statement

The study was approved by the Research Ethics Committee of the School of Public Health of the University of São Paulo (number 4.172.787/2020) and conducted according to the Declaration of Helsinki guidelines.

## Conflicts of Interest

The authors declare no conflicts of interest.

## Supporting information


**Table S1:** Sensitivity analysis of the associations between exposure to exclusive breastfeeding and trajectories of body mass index‐for‐age z‐score (BAZ) and height‐for‐age z‐score (HAZ) in Brazilian children monitored in Primary Health Care, 2015–2019, after inclusion of municipality‐level random effects.

## Data Availability

The data that support the findings of this study were requested from the Brazilian Ministry of Health. Availability of data followed the guidelines for the provision of national databases of health information systems, according to Ordinance No. 884, of December 13, 2011.

## References

[ajhb70315-bib-0001] Baker, P. , J. P. Smith , A. Garde , et al. 2023. “The Political Economy of Infant and Young Child Feeding: Confronting Corporate Power, Overcoming Structural Barriers, and Accelerating Progress.” Lancet 401, no. 10375: 503–524. 10.1016/S0140-6736(22)01933-X. 36764315

[ajhb70315-bib-0002] Balogun, O. O. , A. Dagvadorj , K. M. Anigo , E. Ota , and S. Sasaki . 2015. “Factors Influencing Breastfeeding Exclusivity During the First 6 Months of Life in Developing Countries: A Quantitative and Qualitative Systematic Review.” Maternal & Child Nutrition 11, no. 4: 433–451. 10.1111/mcn.12180. 25857205 PMC6860250

[ajhb70315-bib-0003] Bell, K. A. , C. L. Wagner , H. A. Feldman , R. J. Shypailo , and M. B. Belfort . 2017. “Associations of Infant Feeding With Trajectories of Body Composition and Growth.” American Journal of Clinical Nutrition 106, no. 2: 491–498. 10.3945/ajcn.116.151126. 28659299 PMC5525119

[ajhb70315-bib-0004] Benjamin‐Chung, J. , A. Mertens , J. M. Colford Jr. , et al. 2023. “Early‐Childhood Linear Growth Faltering in Low‐ and Middle‐Income Countries.” Nature 621, no. 7979: 550–557. 10.1038/s41586-023-06418-5. 37704719 PMC10511325

[ajhb70315-bib-0005] Boccolini, C. S. , E. M. A. Lacerda , N. Bertoni , et al. 2023. “Trends of Breastfeeding Indicators in Brazil From 1996 to 2019 and the Gaps to Achieve the WHO/UNICEF 2030 Targets.” BMJ Global Health 8, no. 9: e012529. 10.1136/bmjgh-2023-012529. PMC10481725 37666574

[ajhb70315-bib-0006] Boone‐Heinonen, J. , C. J. Tillotson , J. P. O'Malley , et al. 2019. “Not So Implausible: Impact of Longitudinal Assessment of Implausible Anthropometric Measures on Obesity Prevalence and Weight Change in Children and Adolescents.” Annals of Epidemiology 31: 69–74.e5. 10.1016/j.annepidem.2019.01.006. 30799202 PMC6450088

[ajhb70315-bib-0007] Brasil. Ministério da Saúde . 2023. Guia para a organização da Vigilância Alimentar e Nutricional na Atenção Primária à Saúde [recurso eletrônico]. 1st ed. Universidade Federal de Sergipe.

[ajhb70315-bib-0008] Buccini, G. , J. Pedroso , S. Coelho , et al. 2022. “Nurturing Care Indicators for the Brazilian Early Childhood Friendly Municipal Index (IMAPI).” Maternal & Child Nutrition 18, no. Suppl 2: e13155. 10.1111/mcn.13155. 33945222 PMC8968942

[ajhb70315-bib-0009] Campos, D. S. , and P. C. Fonseca . 2021. “Food and Nutrition Surveillance in 20 Years of the Brazilian National Food and Nutrition Policy. A vigilância Alimentar e Nutricional Em 20 Anos da Política Nacional de Alimentação e Nutrição.” Cadernos de Saúde Pública 37, no. Suppl 1: e00045821. 10.1590/0102-311X00045821. 34730728

[ajhb70315-bib-0010] Carling, S. J. , M. M. Demment , C. L. Kjolhede , and C. M. Olson . 2015. “Breastfeeding Duration and Weight Gain Trajectory in Infancy.” Pediatrics 135, no. 1: 111–119. 10.1542/peds.2014-1392. 25554813 PMC4279065

[ajhb70315-bib-0011] de Souza, G. R. , R. C. Ribeiro‐Silva , M. S. Felisbino‐Mendes , et al. 2023. “Time Trends and Social Inequalities in Infant and Young Child Feeding Practices: National Estimates From Brazil's Food and Nutrition Surveillance System, 2008–2019.” Public Health Nutrition 26, no. 9: 1731–1742. 10.1017/S1368980023001039. 37231823 PMC10478053

[ajhb70315-bib-0012] Dewey, K. G. 1998. “Growth Characteristics of Breast‐Fed Compared to Formula‐Fed Infants.” Biology of the Neonate 74, no. 2: 94–105. 10.1159/000014016. 9691152

[ajhb70315-bib-0013] Dib, S. , F. J. Fair , L. J. McCann , et al. 2024. “Effects of Exclusive Breastfeeding Promotion Interventions on Child Outcomes: A Systematic Review and Meta‐Analysis.” Annals of Nutrition & Metabolism 80, no. 2: 57–73. 10.1159/000535564. 38052180 PMC10997242

[ajhb70315-bib-0014] Diggle, P. J. , P. Heagerty , K. Y. Liang , and S. L. Zeger . 2002. Analysis of Longitudinal Data. 2nd ed. Oxford University Press.

[ajhb70315-bib-0015] Durrleman, S. , and R. Simon . 1989. “Flexible Regression Models With Cubic Splines.” Statistics in Medicine 8, no. 5: 551–561. 10.1002/sim.4780080504. 2657958

[ajhb70315-bib-0016] Gingras, V. , I. M. Aris , S. L. Rifas‐Shiman , K. M. Switkowski , E. Oken , and M. F. Hivert . 2019. “Timing of Complementary Feeding Introduction and Adiposity Throughout Childhood.” Pediatrics 144, no. 6: e20191320. 10.1542/peds.2019-1320. 31757860 PMC6889977

[ajhb70315-bib-0017] Gupta, S. , P. Lal , R. Sharma , A. Gupta , and B. R. Chaudhary . 2025. “The Association Between the Early Introduction of Solid Food and Childhood Obesity Risk: A Systematic Review.” Cureus 17, no. 12: e99245. 10.7759/cureus.99245. 41541930 PMC12801092

[ajhb70315-bib-0018] Heidkamp, R. A. , E. Piwoz , S. Gillespie , et al. 2021. “Mobilising Evidence, Data, and Resources to Achieve Global Maternal and Child Undernutrition Targets and the Sustainable Development Goals: An Agenda for Action.” Lancet 397, no. 10282: 1400–1418. 10.1016/S0140-6736(21)00568-7. 33691095

[ajhb70315-bib-0019] Herber, C. , L. Bogler , S. V. Subramanian , and S. Vollmer . 2020. “Association Between Milk Consumption and Child Growth for Children Aged 6‐59 Months.” Scientific Reports 10, no. 1: 6730. 10.1038/s41598-020-63647-8. 32317668 PMC7174323

[ajhb70315-bib-0020] Horta, B. L. , N. Rollins , M. S. Dias , V. Garcez , and R. Pérez‐Escamilla . 2023. “Systematic Review and Meta‐Analysis of Breastfeeding and Later Overweight or Obesity Expands on Previous Study for World Health Organization.” Acta Paediatrica 112, no. 1: 34–41. 10.1111/apa.16460. 35727183

[ajhb70315-bib-0021] Horta, B. L. , C. G. Victora , G. V. A. França , et al. 2018. “Breastfeeding Moderates FTO Related Adiposity: A Birth Cohort Study With 30 Years of Follow‐Up.” Scientific Reports 8, no. 1: 2530. 10.1038/s41598-018-20939-4. 29416098 PMC5803210

[ajhb70315-bib-0022] Instituto Brasileiro de Geografia e Estatística (IBGE) . 2012. 2010 Brazilian Census [Censo Brasileiro de 2010]. IBGE.

[ajhb70315-bib-0023] Lourenço, B. H. , E. Villamor , R. A. Augusto , and M. A. Cardoso . 2015. “Influence of Early Life Factors on Body Mass Index Trajectory During Childhood: A Population‐Based Longitudinal Analysis in the Western Brazilian Amazon.” Maternal & Child Nutrition 11, no. 2: 240–252. 10.1111/mcn.12005. 23020806 PMC6860355

[ajhb70315-bib-0024] Mertens, A. , J. Benjamin‐Chung , J. M. Colford Jr. , et al. 2023. “Causes and Consequences of Child Growth Faltering in Low‐Resource Settings.” Nature 621, no. 7979: 568–576. 10.1038/s41586-023-06501-x. 37704722 PMC10511328

[ajhb70315-bib-0025] Nelson, S. E. , R. R. Rogers , E. E. Ziegler , and S. J. Fomon . 1989. “Gain in Weight and Length During Early Infancy.” Early Human Development 19, no. 4: 223–239. 10.1016/0378-3782(89)90057-1. 2806151

[ajhb70315-bib-0026] Neves, P. A. R. , G. Gatica‐Domínguez , N. C. Rollins , et al. 2020. “Infant Formula Consumption Is Positively Correlated With Wealth, Within and Between Countries: A Multi‐Country Study.” Journal of Nutrition 150, no. 4: 910–917. 10.1093/jn/nxz327. 31875480 PMC7138652

[ajhb70315-bib-0027] Neves, P. A. R. , J. S. Vaz , F. S. Maia , et al. 2021. “Rates and Time Trends in the Consumption of Breastmilk, Formula, and Animal Milk by Children Younger Than 2 Years From 2000 to 2019: Analysis of 113 Countries.” Lancet. Child & Adolescent Health 5, no. 9: 619–630. 10.1016/S2352-4642(21)00163-2. 34245677 PMC8376656

[ajhb70315-bib-0028] Pérez‐Escamilla, R. , A. Hromi‐Fiedler , E. C. Rhodes , et al. 2022. “Impact of Prelacteal Feeds and Neonatal Introduction of Breast Milk Substitutes on Breastfeeding Outcomes: A Systematic Review and Meta‐Analysis.” Maternal & Child Nutrition 18, no. Suppl 3: e13368. 10.1111/mcn.13368. 35489107 PMC9113480

[ajhb70315-bib-0029] Pérez‐Escamilla, R. , C. Tomori , S. Hernández‐Cordero , et al. 2023. “Breastfeeding: Crucially Important, but Increasingly Challenged in a Market‐Driven World.” Lancet 401, no. 10375: 472–485. 10.1016/S0140-6736(22)01932-8. 36764313

[ajhb70315-bib-0030] Petraroli, M. , E. Castellone , V. Patianna , and S. Esposito . 2021. “Gut Microbiota and Obesity in Adults and Children: The State of the Art.” Frontiers in Pediatrics 9: 657020. 10.3389/fped.2021.657020. 33816411 PMC8017119

[ajhb70315-bib-0031] Putet, G. , J. M. Labaune , K. Mace , et al. 2016. “Effect of Dietary Protein on Plasma Insulin‐Like Growth Factor‐1, Growth, and Body Composition in Healthy Term Infants: A Randomised, Double‐Blind, Controlled Trial (Early Protein and Obesity in Childhood (EPOCH) Study).” British Journal of Nutrition 115, no. 2: 271–284. 10.1017/S0007114515004456. 26586096 PMC4697297

[ajhb70315-bib-0032] Ren, Q. , K. Li , H. Sun , et al. 2022. “The Association of Formula Protein Content and Growth in Early Infancy: A Systematic Review and Meta‐Analysis.” Nutrients 14, no. 11: 2255. 10.3390/nu14112255. 35684055 PMC9183142

[ajhb70315-bib-0033] Rollins, N. C. , N. Bhandari , N. Hajeebhoy , et al. 2016. “Why Invest, and What It Will Take to Improve Breastfeeding Practices?” Lancet 387, no. 10017: 491–504. 10.1016/S0140-6736(15)01044-2. 26869576

[ajhb70315-bib-0034] Roth, D. E. , A. Krishna , M. Leung , J. Shi , D. G. Bassani , and A. J. D. Barros . 2017. “Early Childhood Linear Growth Faltering in Low‐Income and Middle‐Income Countries as a Whole‐Population Condition: Analysis of 179 Demographic and Health Surveys From 64 Countries (1993–2015).” Lancet. Global Health 5, no. 12: e1249–e1257. 10.1016/S2214-109X(17)30418-7. 29132614 PMC5695758

[ajhb70315-bib-0035] Salviano, A. F. , B. M. Guedes , A. A. F. Carioca , S. I. Venancio , G. Buccini , and B. H. Lourenço . 2024. “Positive Changes in Breastfeeding and Complementary Feeding Indicators in Brazil Are Associated With Favorable Nurturing Care Environments.” Public Health 235: 33–41. 10.1016/j.puhe.2024.06.030. 39043006

[ajhb70315-bib-0036] Souza, I. M. , E. M. Araújo , and A. M. D. Silva Filho . 2024. “Incomplete recording of race/colour in health information systems in Brazil: time trend, 2009‐2018. Tendência temporal da incompletude do registro da raça/cor nos sistemas de informação em saúde do Brasil, 2009–2018.” Ciência & Saúde Coletiva 29, no. 3: e05092023. 10.1590/1413-81232024293.05092023. 38451645

[ajhb70315-bib-0037] Srivastava, A. , and J. Swati . 2023. “Appetite Self‐Regulation in Infancy ‐ The Role of Direct Breastfeeding.” World Nutrition 14: 22–27. 10.26596/wn.202314122-27.

[ajhb70315-bib-0038] Stata 17 . 2023. “Version 17.” Accessed June 17, 2023. StataCorp LLC. https://www.stata.com/.

[ajhb70315-bib-0039] Stein, A. D. , M. Wang , R. Martorell , et al. 2010. “Growth Patterns in Early Childhood and Final Attained Stature: Data From Five Birth Cohorts From Low‐ and Middle‐Income Countries.” American Journal of Human Biology: The Official Journal of the Human Biology Council 22, no. 3: 353–359. 10.1002/ajhb.20998. 19856426 PMC3494846

[ajhb70315-bib-0040] Stokes, A. , K. J. Campbell , H. J. Yu , et al. 2021. “Protein Intake From Birth to 2 Years and Obesity Outcomes in Later Childhood and Adolescence: A Systematic Review of Prospective Cohort Studies.” Advances in Nutrition 12, no. 5: 1863–1876. 10.1093/advances/nmab034. 33903896 PMC8483959

[ajhb70315-bib-0041] United Nations . 2020. “The Sustainable Development Goals.” https://www.un.org/sustainabledevelopment/sustainable‐development‐goals/.

[ajhb70315-bib-0042] United Nations Children's Fund, World Health Organization, International Bank for Reconstruction and Development/The World Bank . 2025. Levels and Trends in Child Malnutrition: UNICEF/WHO/World Bank Group Joint Child Malnutrition Estimates: Key Findings of the 2025 Edition. UNICEF and WHO.

[ajhb70315-bib-0043] Verd, S. , G. Ginovart , J. Calvo , J. Ponce‐Taylor , and A. Gaya . 2018. “Variation in the Protein Composition of Human Milk During Extended Lactation: A Narrative Review.” Nutrients 10, no. 8: 1124. 10.3390/nu10081124. 30127252 PMC6115717

[ajhb70315-bib-0044] Victora, C. G. , R. Bahl , A. J. Barros , et al. 2016. “Breastfeeding in the 21st Century: Epidemiology, Mechanisms, and Lifelong Effect.” Lancet 387, no. 10017: 475–490. 10.1016/S0140-6736(15)01024-7. 26869575

[ajhb70315-bib-0045] Wang, J. , Y. Wu , G. Xiong , et al. 2016. “Introduction of Complementary Feeding Before 4months of Age Increases the Risk of Childhood Overweight or Obesity: A Meta‐Analysis of Prospective Cohort Studies.” Nutrition Research 36, no. 8: 759–770. 10.1016/j.nutres.2016.03.003. 27440530

[ajhb70315-bib-0046] World Health Organization . 2006. WHO Child Growth Standards: Length/Heightfor‐Age, Weight‐For‐Age, Weight‐For‐Length, Weight‐Forheight and Body Mass Indexfor‐Age: Methods and Development. WHO.

[ajhb70315-bib-0047] World Health Organization , and United Nations Children's Fund . 2019. Recommendations for Data Collection, Analysis and Reporting on Anthropometric Indicators in Children Under 5 Years Old. WHO and UNICEF.

[ajhb70315-bib-0048] World Health Organization , and United Nations Children's Fund . 2025. Global Breastfeeding Scorecard 2025: Breastfeeding Rates Are Increasing but Improved Support Is Needed. WHO and UNICEF.

[ajhb70315-bib-0049] Wright, C. M. , C. Haig , U. Harjunmaa , H. Sivakanthan , and T. J. Cole . 2022. “Assessing the Optimal Time Interval Between Growth Measurements Using a Combined Data Set of Weights and Heights From 5948 Infants.” Archives of Disease in Childhood 107, no. 4: 341–345. 10.1136/archdischild-2021-322479. 34521634

[ajhb70315-bib-0050] Zheng, M. , N. J. D'Souza , L. Atkins , et al. 2024. “Breastfeeding and the Longitudinal Changes of Body Mass Index in Childhood and Adulthood: A Systematic Review.” Advances in Nutrition 15, no. 1: 100152. 10.1016/j.advnut.2023.100152. 37977327 PMC10714232

