# Supplementary material for: Exclusive Breastfeeding and Growth Trajectories Until 5 Years of Age Among Children Monitored in Primary Health Care in Brazil
Source: Am J Hum Biol. 2026 Jul 20;38(7):e70315. doi: 10.1002/ajhb.70315 (PMC13385645; doi:10.1002/ajhb.70315)
Supplement: Supplementary file 1 — Table S1: Sensitivity analysis of the associations between exposure to exclusive breastfeeding and trajectories of body mass index‐for‐age z‐score (BAZ) and height‐for‐age z‐score (HAZ) in Brazilian children monitored in Primary Health Care, 2015–2019, after inclusion of municipality‐level random effects. [file AJHB-38-e70315-s001.docx]

**Supplementary Table S1.** Sensitivity analysis of the associations between exposure to exclusive breastfeeding and trajectories of body mass index-for-age z-score (BAZ) and height-for-age z-score (HAZ) in Brazilian children monitored in Primary Health Care, 2015-2019, after inclusion of municipality-level random effects.

|  | BAZ | | HAZ | |
| --- | --- | --- | --- | --- |
|  | Exclusive breastfeeding^a^ | | Exclusive breastfeeding^a^ | |
| Age in months | Yes | No | Yes | No |
| Exposure 0 to <3 months |  |  |  |  |
| 3 | 0.27 (0.25; 0.28) | 0.15 (0.14; 0.17) | 0.09 (0.07; 0.11) | -0.06 (-0.08; -0.03) |
| 6 | 0.22 (0.20; 0.24) | 0.21 (0.19; 0.23) | 0.02 (0.00; 0.04) | 0.01 (-0.01; 0.04) |
| 9 | 0.34 (0.32; 0.36) | 0.39 (0.37; 0.41) | -0.07 (-0.09; -0.05) | -0.01 (-0.03; 0.01) |
| 12 | 0.51 (0.49; 0.53) | 0.59 (0.57; 0.61) | -0.16 (-0.18; -0.13) | -0.07 (-0.10; -0.05) |
| 18 | 0.60 (0.58; 0.62) | 0.70 (0.68; 0.72) | -0.23 (-0.25; -0.20) | -0.14 (-0.17; -0.11) |
| 24 | 0.49 (0.46; 0.51) | 0.58 (0.55; 0.60) | -0.21 (-0.24; -0.18) | -0.12 (-0.16; -0.08) |
| 36 | 0.39 (0.36; 0.43) | 0.46 (0.42; 0.51) | -0.09 (-0.14; -0.04) | 0.06 (-0.01; 0.13) |
| 48 | 0.47 (0.41; 0.53) | 0.53 (0.45; 0.61) | 0.07 (-0.03; 0.17) | 0.34 (0.20; 0.48) |
| 60 | 0.58 (0.48; 0.68) | 0.62 (0.49; 0.74) | 0.23 (0.07; 0.40) | 0.64 (0.42; 0.86) |
| Exposure 3 to <6 months |  |  |  |  |
| 6 | 0.31 (0.30; 0.33) | 0.26 (0.24; 0.27) | -0.01 (-0.03; 0.01) | -0.02 (-0.04; 0.00) |
| 9 | 0.43 (0.42; 0.45) | 0.44 (0.42; 0.46) | -0.09 (-0.11; -0.07) | -0.05 (-0.07; -0.03) |
| 12 | 0.52 (0.51; 0.54) | 0.58 (0.57; 0.60) | -0.17 (-0.19; -0.14) | -0.08 (-0.10; -0.05) |
| 18 | 0.57 (0.55; 0.59) | 0.66 (0.64; 0.67) | -0.25 (-0.28; -0.23) | -0.14 (-0.16; -0.12) |
| 24 | 0.51 (0.49; 0.54) | 0.57 (0.55; 0.59) | -0.27 (-0.30; -0.24) | -0.18 (-0.20; -0.15) |
| 36 | 0.43 (0.40; 0.47) | 0.45 (0.42; 0.48) | -0.17 (-0.22; -0.11) | -0.05 (-0.09; -0.01) |
| 48 | 0.40 (0.34; 0.47) | 0.40 (0.35; 0.45) | 0.04 (-0.07; 0.14) | 0.23 (0.15; 0.31) |
| 60 | 0.38 (0.28; 0.48) | 0.37 (0.29; 0.44) | 0.26 (0.09; 0.42) | 0.54 (0.41; 0.66) |

Notes:

BAZ: BMI-for-age z-score; HAZ: height-for-age z-score

Sensitivity analysis models included a municipality-level random intercept. Municipality-level intraclass correlation coefficient (ICC) were 3.8% (BAZ 0 to <3 months, n = 148,705), 3.2% (BAZ 3 to <6 months, n = 135,662), 3.9% (HAZ 0 to <3 months, n = 127,812), and 3.7% (HAZ 3 to <6 months, n = 108,846).

a) Exclusive breastfeeding (EBF): yes (infants 0 to <6 months of age with positive answer to breast milk only, without other liquids or solids); no (infants 0 to <6 months of age with positive responses to water/tea or infant formula or other milks or any complementary solid food, whether or not associated with breast milk).
